# Application of low-coverage whole-genome sequencing technology in risk stratification of colorectal adenomas

**DOI:** 10.3389/fonc.2025.1591548

**Published:** 2025-08-22

**Authors:** Guo Zhili, Xue Yuyue, Yu Fang, Ren Dianqun, Zhang Qin, Liu Jie

**Affiliations:** ^1^ Jiaxing Hospital of Traditional Chinese Medicine, Jiaxing University, Jiaxing, Zhejiang, China; ^2^ Department of Oncology, Putuo Hospital affiliated to Shanghai University of Traditional Chinese Medicine, Shanghai, China

**Keywords:** colorectal adenoma, whole-genome sequencing, tubular adenoma, villous adenoma, chromosomal instability

## Abstract

**Objective:**

The diagnosis of precancerous lesions of colorectal cancer (CRC) presents significant challenges in clinical practice. In this study, we conducted a clinical investigation using the UCAD technique after analyzing chromosomal copy number variations (CNVs) in formalin-fixed, paraffin-embedded (FFPE) samples from various pathological stages, aiming to evaluate the value of detecting chromosomal instability (CIN) in CRC diagnosis.

**Methods:**

Based on colonoscopic pathological findings, we selected 39 FFPE specimens of tubular adenomas, 8 FFPE specimens of villous adenomas, 16 cases diagnosed as tubular-villous adenomas, and 14 cases without defined pathological subtype classification. The UCAD technique was employed to analyze these specimens, with the objective of delineating differences in chromosomal instability among the various pathological subtypes.

**Results:**

UCAD analysis confirmed that among 39 patients diagnosed with tubular adenomas, 12 (30.76%) exhibited CIN positivity, primarily characterized by amplifications of chromosomal segments on 13q, 7, and 8, and losses on 18q and 14q. In the 8 patients diagnosed with villous adenomas, 6 (75%) were CIN-positive, displaying amplifications at 13q, 7, 8q, and 20, along with losses at 18q and 14q. Among 16 patients diagnosed with tubular-villous adenomas, 8 (50%) demonstrated CIN positivity. Additionally, 8 out of 14 cases lacking a defined pathological subtype were CIN-positive.

**Conclusion:**

The assessment of CIN correlates with both pathological subtypes and disease progression. UCAD-based detection of CIN contributes to the diagnosis of colorectal adenomas (CRA), with aberrations in chromosomes 7 and 8 potentially being closely associated with PLCRA.

## 1 Introduction

Colorectal cancer (CRC), as a major global health burden, ranks as the third most common cancer worldwide (1) and poses a serious threat to human life and health. Although its mortality remains high, CRC is generally controllable with early detection and treatment, resulting in a relatively high overall survival rate (2). Colorectal adenoma (CRA) is recognized as the principal precancerous lesion of CRC (3). Patients with CRA have a fourfold higher risk of developing CRC, with approximately 80% of CRC cases originating from CRA (4). Thus, detecting precancerous lesions is an important objective for CRC screening; indeed, both observational and randomized studies have demonstrated that polypectomy during colonoscopy or sigmoidoscopy can effectively prevent CRC (5). However, as colonoscopy is an invasive procedure, it inevitably causes harm to individuals (6). Non-invasive detection methods, such as fecal occult blood tests and carcinoembryonic antigen assays, are less harmful yet suffer from lower accuracy (7–9). Consequently, there is an urgent need for a non-invasive method that accurately diagnoses CRA.

According to Lauren’s criteria, CRA can be subdivided into tubular adenomas and villous adenomas. However, owing to the molecular heterogeneity of CRA, the clinical applicability of these traditional morphology-based classification systems is limited. Therefore, it is imperative to establish a reliable molecular subtyping for CRC to guide clinical practice, determine prognosis, and predict therapeutic response (10).

Combined colonoscopic and histopathological examinations have clear diagnostic significance in the screening of early CRC (11). However, most tissue-based biomarkers for CRC carry a risk of erroneous assumptions, thereby increasing the rate of missed diagnoses due to tumor heterogeneity. In addition to microbial infections, alterations in genomic stability play a critical role as driving forces in CRA. Notably, chromosomal instability (CIN), often described as somatic copy number alterations (SCNAs) (12) accompanied by focal oncogene amplifications or tumor suppressor gene deletions, is one of the most common types of genetic alterations. Data from the TCGA database indicate that CRA can be classified into two distinct subtypes based on the presence or absence of SCNAs. Previous studies using array comparative genomic hybridization have demonstrated that the two CRA subtypes—high-CIN and low-CIN—exhibit distinct gene expression profiles and survival outcomes (13–15), underscoring the prognostic value of CIN in CRA.

Nevertheless, array comparative genomic hybridization is expensive and technically complex, thereby limiting its clinical utility (16). In 2014, low-coverage whole-genome sequencing (LC-WGS) was developed as a simple, cost-effective, and reliable technique for identifying SCNAs in tumors. Accordingly, the aim of this study is to develop a mature and economical molecular subtyping method for CRA using LC-WGS, in order to identify the driving factors of colorectal tumorigenesis and to provide a foundation for CRC risk stratification and targeted therapy (17, 18).

Chromosomal instability (CIN) is characterized by the persistent aberrant segregation of chromosomes in cancer cells relative to normal cells, primarily manifesting as somatic copy number abnormalities accompanied by focal oncogene amplifications or tumor suppressor gene deletions. CIN represents one of the predominant forms of genomic instability in various human cancers; it is present in most solid malignancies and lies at the core of cancer evolution. As an emerging alternative diagnostic tool and a driver of tumorigenesis, CIN has been shown to affect tumor initiation and progression by promoting intratumoral heterogeneity, inducing spatial and temporal diversification of tumor subclones, enhancing metastasis, accelerating tumor phenotypic adaptation, facilitating cellular immortalization, enabling immune evasion, and conferring drug resistance (19).

The Cancer Genome Atlas (TCGA) encompasses the full spectrum of genomic features in human cancers. We leveraged this database to investigate the impact of CIN on CRC by analyzing chromosomal copy number variations, thereby confirming the characteristics of CIN associated with CRC and correlating these chromosomal features with clinical data (20).

In this study, we employed an ultra-sensitive chromosomal aneuploidy detection (UCAD) technique using genomic DNA extracted from colonic mucosal tissue, combined with LC-WGS and bioinformatics analysis, to assess chromosomal instability at the whole-genome level—thus achieving both qualitative and quantitative evaluations of chromosomal stability. This approach holds significant promise for facilitating early cancer diagnosis, preventing progression at later stages, enabling early intervention, and aiding prognostic assessments. Furthermore, the UCAD-based analysis of CIN provides a novel, as yet underexplored, avenue for determining and predicting the severity and prognosis of CRA patients. Therefore, we conducted this study to evaluate the diagnostic and prognostic value of CIN in identifying colorectal precancerous lesions.

## 2 Materials and methods

### 2.1 Patient characteristics and ethical statement

FFPE samples were collected from 77 patients who underwent colonoscopy to assess the risk of colorectal adenoma with follow-up data recorded until May 2024. This study was approved by the Ethics Committee of Zhejiang Jiaxing Traditional Chinese Medicine Hospital (Approval No.: Jia TCM Ethics 2023 Research No. 063) (**Table 1**).

**Table 1:** Clinical features of the patients.

Patients (n=77, N/A=14)	Characteristics	Tubular	Villous	Tubulovillous	P value
		n=39	n=8	n=16	
Age					0.066
	<60	27 (69.2%)	2 (25.0%)	9 (56.3%)	
	≥60	12 (30.8%)	6 (75.0%)	7 (43.7%)	
Sex					0.197
	Male	25 (64.1%)	7 (87.5%)	8 (50.0%)	
	Female	14 (35.9%)	1 (12.5%)	8 (50.0%)	
Diabetes history					0.050
	Yes	0 (0.0%)	1 (12.5%)	2 (12.5%)	
	No	39 (100.0%)	7 (87.5%)	14 (87.5%)	
Coronary heart disease history					0.141
	Yes	0 (0.0%)	1 (12.5%)	1 (6.3%)	
	No	39 (100.0%)	7 (87.5%)	15 (93.7%)	

### 2.2 DNA extraction

DNA was extracted from formalin-fixed paraffin-embedded (FFPE) samples utilizing the QIAseq cfDNA Extraction Kit (Qiagen, 69504). A total of 10 ng of cfDNA was subsequently employed for sequencing library preparation using the NEBnext Ultra II FS DNA Library Prep Kit. The DNA fragments were tagged with 8 bp-barcoded sequencing adapters and amplified through PCR.

### 2.3 Low−coverage whole−genome sequencing

Purified sequencing libraries were massively sequenced by Illumina HiSeq Xten platform using 150-base paired-end reads across a single lane, providing efficient and high-throughput low-pass sequencing. Segmental copy numbers were determined using a bespoke analytical pipeline, the ultrasensitive chromosomal aneuploidy detector (UCAD), enabling precise detection of chromosomal copy number variations.

Samples were excluded if the absolute median deviation of the copy ratio (log ratio) between adjacent bins across the genome exceeded 0.38, indicating poor sequence quality. Reads were aligned to the EBV reference genome (gi|82503188). Matches with no more than one mismatch were counted as EBV. Similarly, Helicobacter pylori was detected using the same approach with reference genome gi|261838873. Samples with more than four EBV reads were labeled as EBV tumor samples. Samples with more than four *Helicobacter pylori* reads were labeled as *Helicobacter pylori* tumor samples.

### 2.4 UCAD and LC-WGS testing

Agarose gel electrophoresis was used to analyze DNA degradation and RNA contamination. DNA purity was measured using a nanodrop spectrophotometer (OD260/280 ratio), and DNA concentration was accurately quantified with Qubit. Whole-genome DNA was fragmented into small portions either physically or enzymatically. Library construction involved adding adapters to the fragmented sequencing pieces. DNA fragments underwent end-repair to create blunt ends. Adapters were added, U-shaped adapters were converted to Y-shaped adapters, and impurities were removed via magnetic bead purification. PCR amplification was performed to incorporate indexes and two oligonucleotides complementary to the sequencing chip. A second magnetic bead purification step was conducted to remove residual polymerases and other impurities. Final quality control was performed, including DNA concentration measurement, agarose gel electrophoresis, and fragment length analysis, to complete library construction. Library fragments served as templates for DNA replication in bridge amplification and single-base extension sequencing.

### 2.5 Statistical analyses and data visualization

DNA from mucosal tissues was extracted and analyzed using the Illumina X10 platform, ensuring high-quality sequencing data. Approximately A minimum of 10 million paired-end reads were generated for each sample and subsequently aligned to the human reference genome (hg19) using BWA version 0.7.17-r1188. Genomic coverage was assessed using the mpileup software package, enabling precise read depth quantification. The average coverage for each 200-kilobase (kb) bin was calculated, and Z-scores for each bin were normalized using the following formula:


$$Z_{bin}=coverage_{normalized}=\frac{coverage_{raw}-mean\left(coverage_{controls,\;raw}\right)}{\begin{array}{c} stdev\left(coverage_{controls,raw}\right)\; \end{array}}$$

*Z_bin_*: Standardized Z-score for a specific genomic bin;
*coverage_raw_*: aw coverage value of the bin under investigation;
*coverage_controls, raw_*: Raw coverage values from control samples;
*mean(coverage_controls, raw_)*: Mean raw coverage value across control samples;
*stdev(coverage_controls, raw_)*: Standard deviation of raw coverage values in control samples.

The Circular Binary Segmentation (CBS) algorithm, implemented in the R package DNAcopy (Seshan and Olshen, 2013), was employed to identify significant genomic breakpoints and define copy number segments.

A P value of< 0.05 was considered to denote a statistically significant binary segmentation. The absolute segment value was used for further analysis. The sensitivity and specificity of UCAD were estimated by receiver operating characteristic curves. For categorical variables, the chi-square test was employed. All statistical analyses were performed using SPSS17.0 (IBM, Foster City, CA, United States). The anonymized data and R code used in the statistical analysis will be made available on request. Microsatellite instability analysis is performed by MILO (https://github.com/QingliGuo/MILO) analysis.

Statistical analyses were performed using R software, version 3.4.3 (R Foundation for Statistical Computing). Anonymized data and R code used in the statistical analysis will be made available on request.

## 3 Results

**Figure 1:**
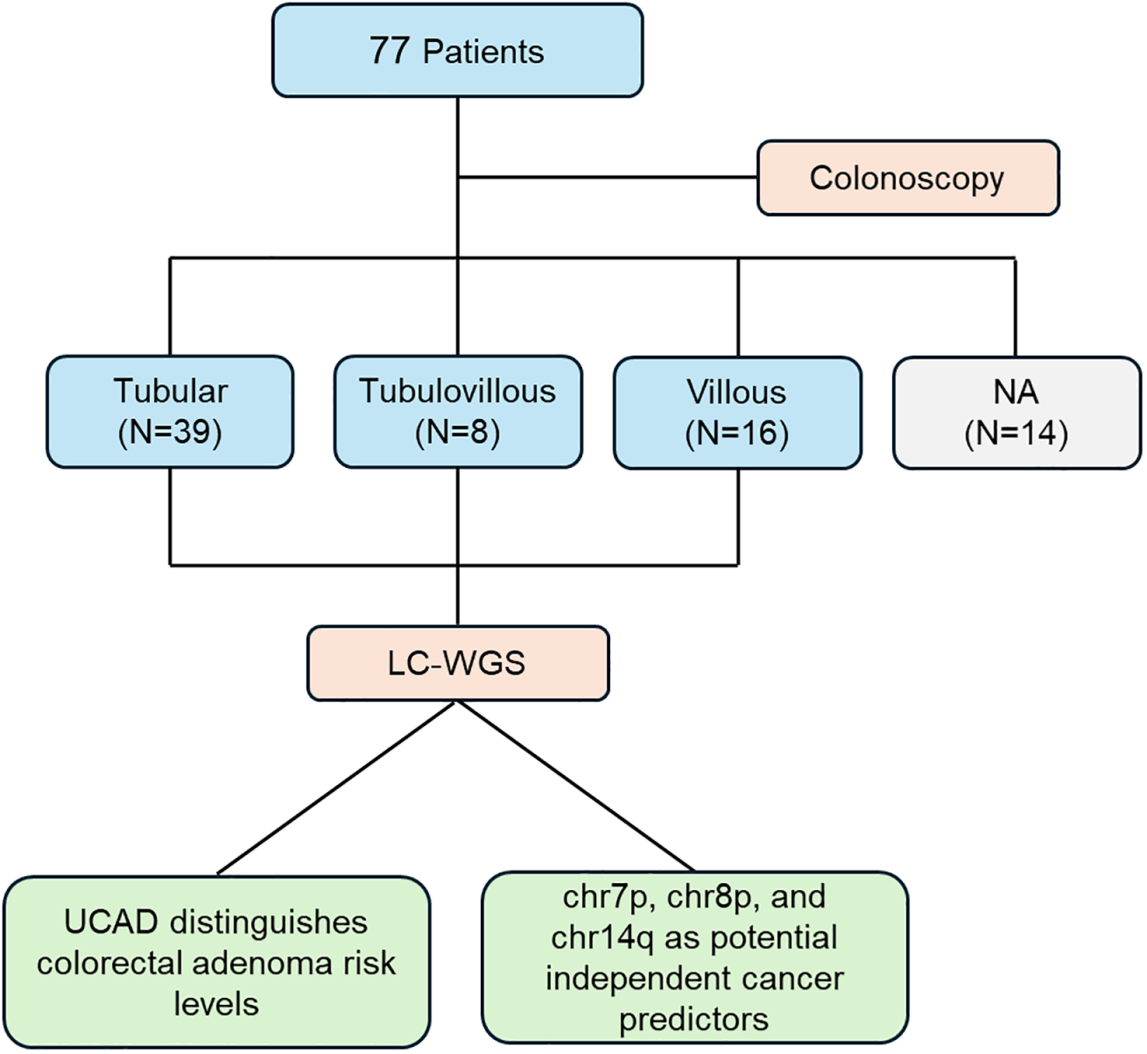
Flowchart of colorectal adenoma patient categorization and analysis.

This study involved 77 patients who underwent colonoscopy to evaluate the risk of colorectal adenoma. Based on histopathological assessment, patients were divided into three subgroups: tubular adenoma (N=39), tubular-villous adenoma (N=8), and villous adenoma (N=16), while an additional subgroup (N=14) lacked an available classification (NA). Following sample collection, low-coverage whole-genome sequencing (LC-WGS) was performed to investigate genomic variations. The primary findings include that the UCAD method effectively distinguishes different risk levels of colorectal adenoma, providing a potential tool for risk stratification. Chromosomal alterations on chr7p, chr8p, and chr14q emerged as independent predictors of cancer risk, indicating their potential utility in early detection and prognostication. This workflow, **Figure 1**, underscores the integration of genomic analysis with clinical evaluation, contributing to a deeper understanding of colorectal adenoma progression and its association with malignant tumor risk.

### 3.1 Patient characteristics

A total of 77 FFPE samples were collected. All samples passed QC and were included in this study (**Table 1**). A total of 77 CRA patients were analyzed. The median age was 57 years, although subtype-specific differences were noted: patients with tubular adenomas had a median age of 54 years, significantly lower than that of patients with villous adenomas (median 63 years). Patients in the villous and tubulovillous groups tended to be older compared to those in other groups. Males predominated in the villous adenoma group (87.5%), compared to 64.1% in the tubular adenoma group. Baseline characteristics of these patients are summarized in **Table 1**.

### 3.2 Differences in chromosomal copy number variations

**Figure 2:**
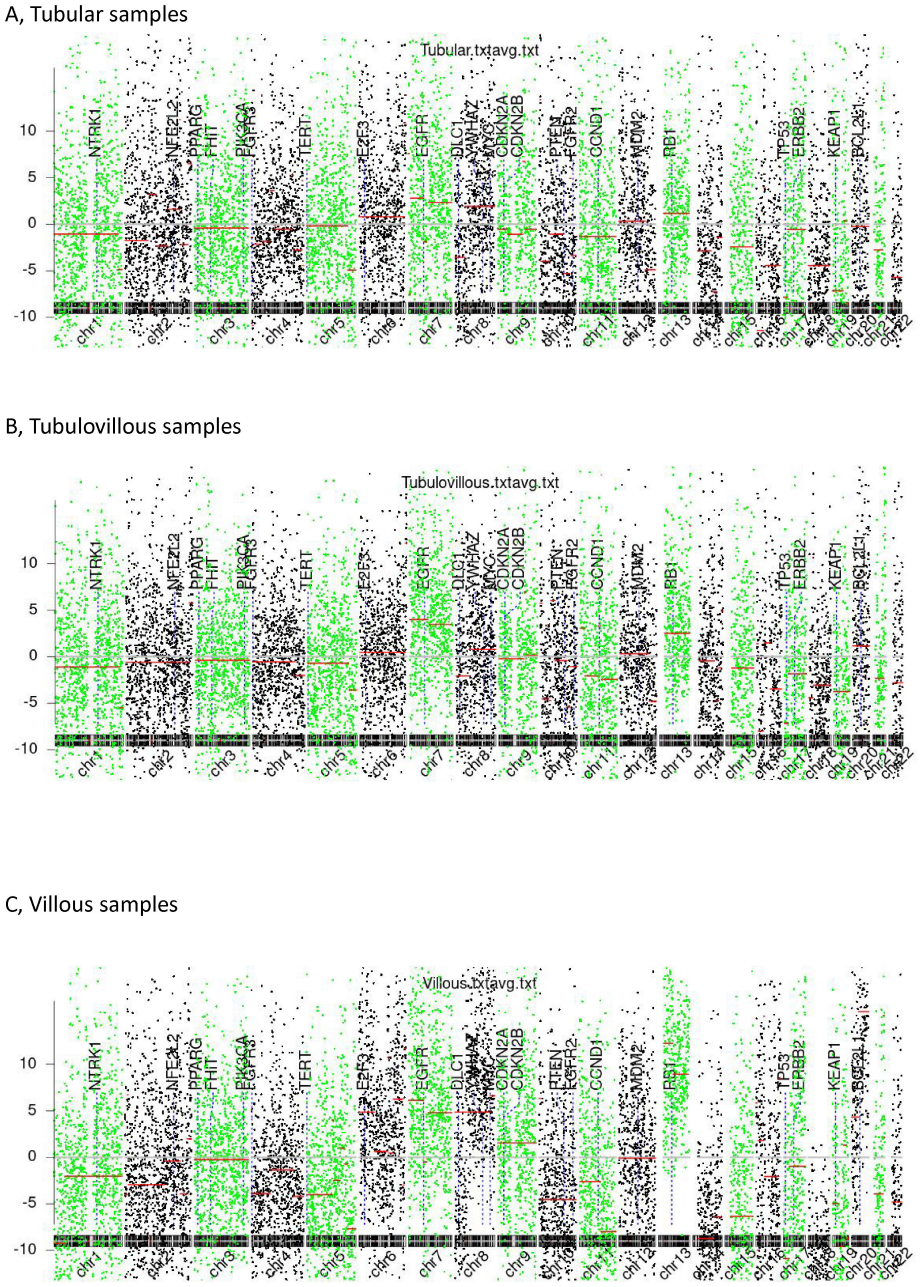
Chromosomal instability (CIN). **(A)** Tubular samples, **(B)** Tubulovillous samples, **(C)** Villous samples.

In **Figure 2**, we summarize the observed genome-wide copy number alterations. Interestingly, we found that chromosomal arm imbalances were driven by breakpoints located at the centromeres, revealing an increasing degree of chromosomal instability across tubular, tubulovillous, and villous samples. Tubular samples exhibited the lowest degree of dispersion, suggesting relative genomic stability. In contrast, villous samples showed the highest level of dispersion, indicating pronounced chromosomal instability, which may correlate with their higher aggressiveness and poorer prognosis.

### 3.3 Diagnostic performance

Among the 39 patients with a histopathological diagnosis of tubular adenoma, 12 (30.76%) were CIN-positive, primarily exhibiting amplifications of chromosomal fragments on chromosomes 7 and 8 (**Table 2**). In the 8 patients with a histopathological diagnosis of villous adenoma FFPE specimens, 6 (75%) were CIN-positive, also mainly showing amplifications on chromosomes 7 and 8. Among the 16 patients with a histopathological diagnosis of tubular-villous adenoma FFPE specimens, 8 (50%) were CIN-positive. Additionally, within the subgroup of 14 cases lacking a defined pathological classification, 8 cases were CIN-positive.

**Table 2:** The diagnostic efficacy of CIN in the three categories: Tubular samples, Tubulovillous samples, and Villous samples.

Pathological Type	Unstable (CIN+)	Stable (CIN-)	P-value
Tubular	12	27	0.049
Villous	6	2	
Tubulovillous	8	8	
N/A	6	8	

### 3.4 Chromosomal and gene copy number instability

**Figure 3:**
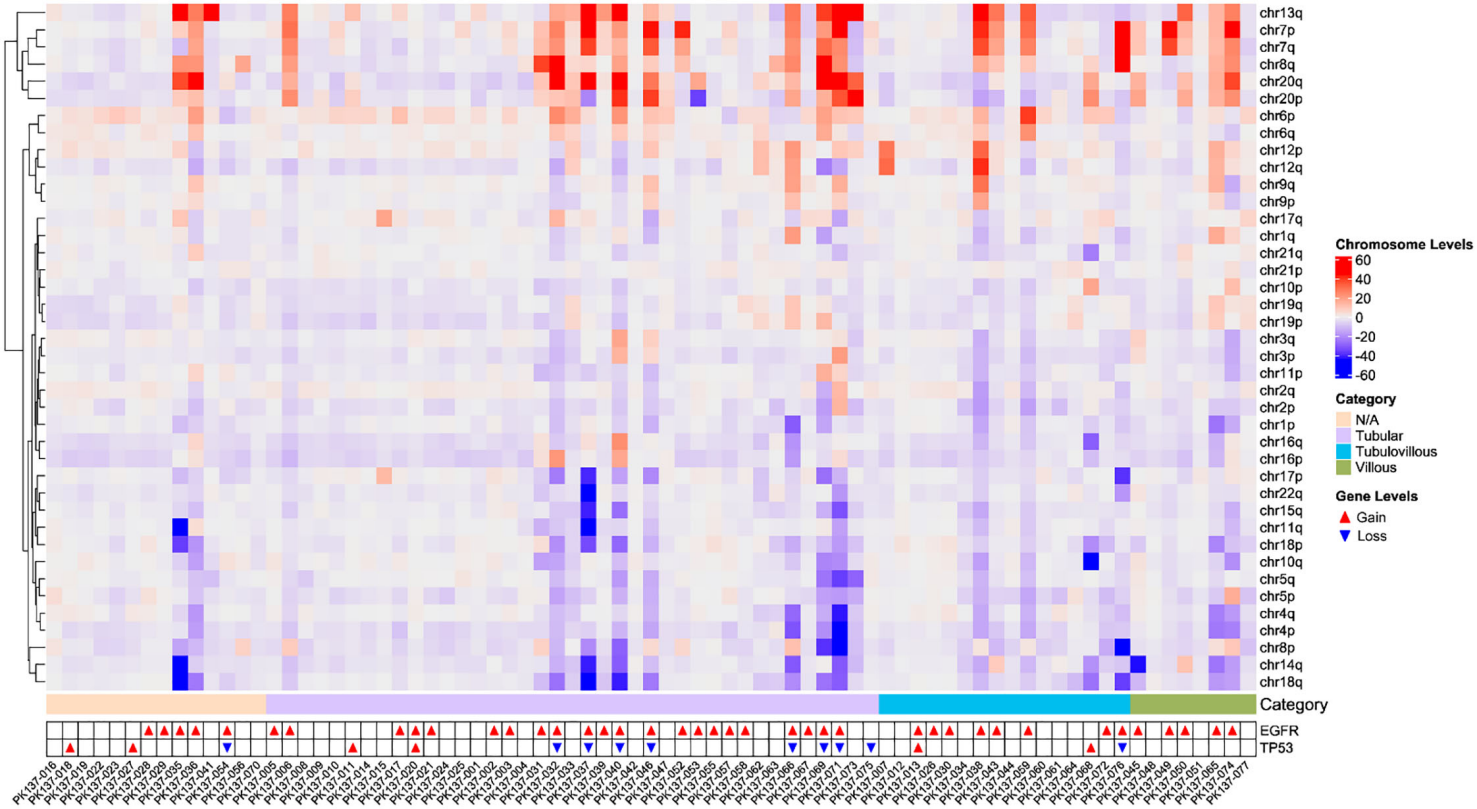
The heatmap of chromosomal copy number instability in all patients, including the gain and loss status of EGFR and TP53.

As depicted in **Figure 3**, CIN-positive patients exhibited significant chromosomal copy number amplifications on chr13q, chr7, chr8q, chr20, and chr6, while notable copy number losses were observed on chr18q, chr14q, chr8p, chr4, and chr5. Among these, compared to other patients, villous adenoma samples exhibited more frequent amplifications or losses of chromosomal arms. In most samples (52.95%), EGFR amplification was observed, which may lead to abnormal activation of the MAPK/PI3K pathways, contributing to increased cell proliferation and oncogenesis; this phenomenon reached 87.5% in villous adenoma patients. MYC amplification was also prevalent, with an overall occurrence rate of 50.65%, accounting for 71.79% in tubular adenoma patients and reaching 75% in villous adenoma patients. SMAD4 and DCC primarily exhibited copy number losses. Loss of SMAD4 suggests impairment of the TGF-β-mediated growth inhibitory pathway, potentially accelerating oncogenesis; notably, the loss rate of SMAD4 was relatively high (75%) in villous adenoma patients. DCC may affect apoptotic processes and cell adhesion molecule function.

### 3.5 Microsatellite instability

**Figure 4:**
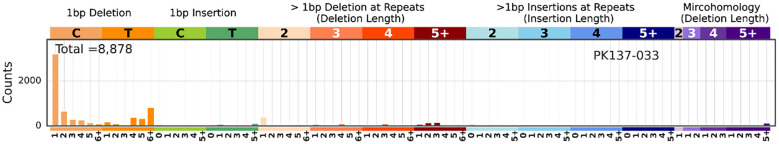
The MSI analysis results generated by MILO and SigProfilerMatrixGenerator. In the x-axis, each category is further subdivided based on the repeat length (e.g., 1, 2, 3, 4, 5, 6+). The y-axis represents the mutation counts. Among all the samples, MSI was detected in PK137-033. The rest of the results are in the **Supplementary Table**.

MSI and its associated features, such as long deletions, provide insights into the progression of DNA instability, which is critical in the adenoma-carcinoma sequence. Regular MSI monitoring could aid in stratifying patients by risk and guiding personalized interventions. **Figure 4** illustrates that mutations in longer repeat regions (≥5 bp) show a striking increase, particularly for deletions, as highlighted in the red section of the figure. This distinct pattern of long repeat deletions is a hallmark of MSI, indicating a high mutation burden in the analysed samples. The striking increase in deletions in regions with longer repeats (≥5 bp) suggests the accumulation of replication slippage errors that are typical in MSI-high tumours. Normally, such errors are corrected by the mismatch repair (MMR) system.

## 4 Discussion

Colorectal cancer (CRC) exhibits high incidence and mortality rates in our country, with a multifactorial etiology. Genetic factors, epigenetic modifications, and the regulation of numerous related genes and chromosomes contribute to the complex heterogeneity of tumors (21). This disease involves multiple genes with distinct genetic characteristics at various stages. The genetic, individual, and molecular complexities of colorectal adenoma (CRA) necessitate its characterization via gene panels or clustering methods (22). Molecular subtyping of CRA involves screening for genes or protein markers associated with tumorigenesis, diagnosis, and prognosis. Current studies have focused on oncogenes, tumor suppressor genes, cell adhesion molecules, growth factors, and certain hormone receptors. Although most of these markers exhibit suboptimal sensitivity, specificity, or reliability, a few have been recognized as effective biomarkers. Colonic adenocarcinoma—the most common form of CRC—displays significant heterogeneity among patients due to its inherent aggressiveness, leading to high mortality. Despite recent advances in diagnosis and treatment, the 5-year overall survival (OS) remains poor (23, 24). Traditional morphology-based classification systems, such as the Lauren classification (intestinal, diffuse, and mixed types) and the WHO classification (papillary, tubular, and mucinous types), are used. For predicting lymph node metastasis risk, an improved WHO classification can divide CRAs into differentiated and undifferentiated types. However, dysregulation of oncogenes and tumor suppressor genes induced by various genetic and epigenetic alterations has been demonstrated in multiple studies as a critical driving force for tumorigenesis. Currently, morphology-based clinical subtyping of CRA neither captures its molecular heterogeneity nor guides the prediction of prognosis or treatment response in advanced CRA patients. Although molecular subtyping may add complexity to the classification, defining specific CRA subtypes based on molecular and genetic features is essential for precise and selective targeted anti-cancer therapy (1).

Approximately 40% of CRAs exhibit features of high chromosomal instability (CIN). Notably, ERBB2 amplification was relatively common in this study. HER2, also known as ERBB2, is a member of the ERBB protein family, which includes EGFR (or HER1), HER3, and HER4. Trastuzumab, a humanized monoclonal antibody, specifically binds to HER2, inhibiting its homodimerization and phosphorylation, thereby suppressing the proliferation of tumor cells that overexpress HER2 (2).

The LC-WGS-based UCAD assay is estimated to cost approximately 50 USD per patient. With the rapid decline in next-generation sequencing costs, it is anticipated that UCAD will become even more cost-effective in the near future. Traditionally, multiple tests—including copy number variation analyses (most of which employ FISH techniques, such as HER2 FISH)—are performed separately, imposing a significant financial burden on CRC patients. Moreover, owing to the practicality of whole-genome sequencing (WGS), the UCAD assay is capable of capturing not only human DNA but also microbial DNA, thereby providing more comprehensive information for CRA subtyping compared to other methods. Overall, this new technology can guide precise CRA treatment in a more economical manner (3).

It is particularly important to identify effective screening methods during the precancerous stages of CRC. The continuous advancement of high-throughput sequencing technologies has enabled comprehensive, multi-level investigations of tumors at both the genomic and transcriptomic levels. Integrating available multi-omics data with clinical patient information is more conducive to identifying effective therapeutic targets and prognostic indicators (4).

CIN is a phenotype in which cancer cells, compared to normal cells, exhibit instability of DNA or structural chromosomes (S-CIN). CIN is considered one of the most fundamental causes of cancer development and is present in nearly all malignant tumors. During mitosis, when cancer cells experience uneven distribution of chromosomes in daughter cells and this erroneous segregation persists, it results in changes in chromosomal copy numbers or amplification/deletion of internal chromosomal segments (5). CIN acts as a driving force for tumorigenesis, meaning that once chromosomal instability occurs in certain regions of the body, cancer has either already developed or is imminent. Studies have shown that CIN influences tumor initiation and progression by driving intratumoral heterogeneity, inducing spatial and temporal diversification of tumor subclones, promoting metastasis, accelerating tumor phenotypic adaptation, conferring cellular immortality, enabling immune evasion, and fostering drug resistance. In this study, the rate of CIN positivity exhibited a significant gradient across adenoma subtypes (villous: 75% > mixed: 50% > tubular: 30.76%), indicating that CIN may serve as a molecular yardstick for precancerous lesions—particularly given the high-frequency amplification of 7p and 8q and deletion of 14q.

In this study, the detection rate of 14q deletion in villous adenomas reached as high as 75%, which is significantly higher than that in tubular adenomas (31%). Loss of 14q can impair a cell’s ability to perform homologous recombination repair. This chromosomal region also harbors two key tumor suppressor genes, DCC (located at 14q32.3) and SMAD4 (located at 14q22.2). The 14q arm is rich in cancer-related genes, and its 32.2–32.3 segment has been identified as the second largest tumor suppressor gene cluster in the human genome (second only to the TP53 region on 17p). Whole-genome sequencing data reveal that this chromosome contains structural fragile sites, such as FRA14A (14q24.3) and FRA14C (14q32.1), which are associated with a predisposition to double-strand breaks under DNA replication stress; GEO data analysis further indicates that the 14q32 region exhibits hypermethylation in CRC, potentially silencing downstream tumor suppressor genes. These alterations contribute to the continuous accumulation of CIN.

In various cancer models, chromosome 7 is frequently reported to contain regions that have undergone genetic alterations or exhibit inherent instability (6). However, since most previous studies have utilized intermediate chromosomes or BAC array CGH, there has been no systematic search for individual genes that experience copy number gains or amplifications. Human chromosome 7 is approximately 159 Mb in length and harbors 1,150 genes along with 940 pseudogenes, many of which are implicated in various human diseases including cystic fibrosis, deafness, B-cell lymphoma, and cancer. This chromosome contains well-known oncogenes that demonstrate gene amplification, such as the epidermal growth factor receptor (EGFR, located at 7p12), hepatocyte growth factor (HGF, at 7q21.1), and the MET proto-oncogene (met/HGFR, at 7q31). Since gene amplification is one of the most common mechanisms of oncogenic activation, it is crucial to identify the complete repertoire of potentially amplified genes in tumor tissues within a given cancer model. Among these, EGFR – the protein product of the HER-1 proto-oncogene – is a key oncogene in colorectal cancer whose receptor tyrosine kinase activity triggers essential signaling pathways for tumor cell growth and survival (7).

The classical adenoma–carcinoma sequence involves the accumulation of stepwise somatic mutations and copy number alterations affecting major oncogenes and tumor suppressor genes. Among these, APC mutation is considered the gate keeper event in early adenoma formation, leading to aberrant activation of the WNT/β-catenin pathway (25). KRAS mutations typically follow and promote cellular proliferation by activating the RAS–RAF–MEK–ERK cascade. TP53 inactivation occurs at later stages and disrupts DNA damage response and apoptotic control. In parallel, loss of SMAD4, located at 18q21 or sometimes 14q22 depending on cytogenetic context, impairs TGF-β–mediated growth inhibition and is frequently observed in advanced adenomas or intramucosal carcinomas (26).

Additionally, other critical molecular alterations include PIK3CA mutations (PI3K pathway activation), FBXW7 mutations (cell cycle dysregulation), and MYC amplification (observed in our cohort at chr8q24), which is strongly associated with proliferative signaling (27). Amplification of EGFR (chr7p12) and ERBB2 (HER2) also supports sustained mitogenic stimulation and is a prominent feature in our CIN-positive patients. These events, in combination with increasing chromosomal instability, drive malignant transformation (28).

Chromosome 8 is a medium-sized autosome in humans that exhibits an exceptionally high mutation rate due to positive selection. Telomere shortening on this chromosome may represent a mechanism that promotes the development of chromosomal instability in the context of aging and chronic diseases. This relatively high level of genomic instability on chromosome 8 has been observed not only throughout evolution but also in various mutational diseases such as tumorigenesis and subsequent invasion/metastasis. Amplification of the 8q arm is closely associated with the intestinal type of colorectal cancer. One study employing comparative genomic hybridization (CGH) assessed DNA copy number alterations (CNAs) in 53 tumors, correlating these alterations with clinicopathological features and TP53 status, and found 8q abnormalities in 43% of cases. Moreover, a large-scale label-free quantitative proteomics study identified defects in the 8p21-p23 region during the development of digestive organ tumors (8). It has been demonstrated that amplification of chromosome 8 leads to high expression of the MYC proto-oncogene. c-Myc plays a critical role in tumor development by coordinating gene expression across various human cancers, and its aberrant expression is a key driver of colorectal cancer progression. One of the c-Myc genes is located at 8q24.2–3 and encodes a nuclear transcription factor that regulates cell proliferation, differentiation, and apoptosis. Furthermore, analyses of the karyotype and phenotype of circulating tumor cells (CTCs) in patients with advanced colorectal cancer (ACRA) have shown that CTCs with different ploidies of chromosome 8 are associated with differential sensitivity or resistance to chemotherapeutic agents. Consequently, amplification of chromosome 8 may further promote tumor development.

Our findings indicate that gains on chromosome 7p (chr7p+), gains on chromosome 8p (chr8p+), and losses on chromosome 14q (chr14q-) may serve as independent predictors of cancer. The UCAD assay may represent an alternative, non-invasive biomarker for cancer prediction. Although our data are highly informative, the relatively small sample size necessitates large-scale prospective clinical trials to further validate the reliability of these results.

## Data Availability

The original contributions presented in the study are included in the article/**Supplementary Material**. Further inquiries can be directed to the corresponding author.

## References

[1] Li J Ma X Chakravarti D Shalapour S DePinho RA. Genetic and biological hallmarks of colorectal cancer. Genes Dev. (2021) 35:787–820. doi: 10.1101/gad.348226.120, PMID: 34074695 PMC8168558

[2] Baidoun F Elshiwy K Elkeraie Y Merjaneh Z Khoudari G Sarmini MT. Colorectal Cancer Epidemiology: Recent Trends and Impact on Outcomes. Curr Drug Targets. (2021) 22:998–1009. doi: 10.2174/18735592MTEx9NTk2y, PMID: 33208072

[3] Mahmoud NN. Colorectal Cancer: Preoperative Evaluation and Staging. Surg Oncol Clin N Am. (2022) 31:127–41. doi: 10.1016/j.soc.2021.12.001, PMID: 35351269

[4] Eng C Jácome AA Agarwal R Hayat MH Byndloss MX Holowatyj AN. A comprehensive framework for early-onset colorectal cancer research. Lancet Oncol. (2022) 23:e116–28. doi: 10.1016/S1470-2045(21)00588-X, PMID: 35090673

[5] Zhou E Rifkin S. Colorectal Cancer and Diet: Risk Versus Prevention, Is Diet an Intervention? Gastroenterol Clin North Am. (2021) 50:101–11. doi: 10.1016/j.gtc.2020.10.012, PMID: 33518157

[6] Yan H Talty R Johnson CH. Targeting ferroptosis to treat colorectal cancer. Trends Cell Biol. (2023) 33:185–8. doi: 10.1016/j.tcb.2022.11.003, PMID: 36473802

[7] Hollis RH Chu DI. Healthcare Disparities and Colorectal Cancer. Surg Oncol Clin N Am. (2022) 31:157–69. doi: 10.1016/j.soc.2021.11.002, PMID: 35351271 PMC8968072

[8] Klimeck L Heisser T Hoffmeister M Brenner H. Colorectal cancer: A health and economic problem. Best Pract Res Clin Gastroenterol. (2023) 66:101839. doi: 10.1016/j.bpg.2023.101839, PMID: 37852707

[9] Sakthianandeswaren A Parsons MJ Mouradov D Sieber OM. *MACROD2*deletions cause impaired PARP1 activity and chromosome instability in colorectal cancer. Oncotarget. (2018) 9:33056–8. doi: 10.18632/oncotarget.25887, PMID: 30237848 PMC6145690

[10] Pino MS Chung DC. The chromosomal instability pathway in colon cancer. Gastroenterology. (2010) 138:2059–72. doi: 10.1053/j.gastro.2009.12.065, PMID: 20420946 PMC4243705

[11] Heiser CN Simmons AJ Revetta F McKinley ET Ramirez-Solano MA Wang J. Molecular cartography uncovers evolutionary and microenvironmental dynamics in sporadic colorectal tumors. Cell. (2023) 186:5620–5637.e16. doi: 10.1016/j.cell.2023.11.006, PMID: 38065082 PMC10756562

[12] Li J Hubisz MJ Earlie EM Duran MA Hong C Varela AA. Non-cell-autonomous cancer progression from chromosomal instability. Nature. (2023) 620:1080–8. doi: 10.1038/s41586-023-06464-z, PMID: 37612508 PMC10468402

[13] Cisyk AL Nugent Z Wightman RH Singh H McManus KJ. Characterizing Microsatellite Instability and Chromosome Instability in Interval Colorectal Cancers. Neoplasia. (2018) 20:943–50. doi: 10.1016/j.neo.2018.07.007, PMID: 30121009 PMC6098200

[14] Malki A ElRuz RA Gupta I Allouch A Vranic S Al Moustafa AE. Molecular Mechanisms of Colon Cancer Progression and Metastasis: Recent Insights and Advancements. Int J Mol Sci. (2020) 22:130. doi: 10.3390/ijms22010130, PMID: 33374459 PMC7794761

[15] Baergen AK Jeusset LM Lichtensztejn Z McManus KJ. Diminished Condensin Gene Expression Drives Chromosome Instability That May Contribute to Colorectal Cancer Pathogenesis. Cancers (Basel). (2019) 11:1066. doi: 10.3390/cancers11081066, PMID: 31357676 PMC6721357

[16] Mouradov D Greenfield P Li S In EJ Storey C Sakthianandeswaren A. Oncomicrobial Community Profiling Identifies Clinicomolecular and Prognostic Subtypes of Colorectal Cancer. Gastroenterology. (2023) 165:104–20. doi: 10.3390/cancers11081066, PMID: 36933623

[17] Martin S Scorzoni S Cordone S Mazzagatti A Beznoussenko GV Gunn AL. A p62-dependent rheostat dictates micronuclei catastrophe and chromosome rearrangements. Science. (2024) 385:eadj7446. doi: 10.1126/science.adj7446, PMID: 39208097 PMC11664475

[18] Cornish AJ Gruber AJ Kinnersley B Chubb D Frangou A Caravagna G. The genomic landscape of 2,023 colorectal cancers. Nature. (2024) 633:127–36. doi: 10.1038/s41586-024-07747-9, PMID: 39112709 PMC11374690

[19] Cisyk AL Penner-Goeke S Lichtensztejn Z Nugent Z Wightman RH Singh H. Characterizing the prevalence of chromosome instability in interval colorectal cancer. Neoplasia. (2015) 17:306–16. doi: 10.1016/j.neo.2015.02.001, PMID: 25810015 PMC4372653

[20] Shin G Greer SU Hopmans E Grimes SM Lee H Zhao L. Profiling diverse sequence tandem repeats in colorectal cancer reveals co-occurrence of microsatellite and chromosomal instability involving Chromosome 8. Genome Med. (2021) 13:145. doi: 10.1186/s13073-021-00958-z, PMID: 34488871 PMC8420050

[21] Fang X Yu WY Zhu CM Zhao N Zhao W Xie TT. Chromosome instability functions as a potential therapeutic reference by enhancing chemosensitivity to BCL-XL inhibitors in colorectal carcinoma. Acta Pharmacol Sin. (2024) 45:2420–31. doi: 10.1038/s41401-024-01372-y, PMID: 39187678 PMC11489767

[22] Chen B Dragomir MP Fabris L Bayraktar R Knutsen E Liu X. The Long Noncoding RNA CCAT2 Induces Chromosomal InstabilityThrough BOP1-AURKB Signaling. Gastroenterology. (2020) 159:2146–2162.e33. doi: 10.1053/j.gastro.2020.08.018, PMID: 32805281 PMC7725986

[23] Voutsadakis IA. Chromosome 20q11.21 Amplifications in Colorectal Cancer. Cancer Genomics Proteomics. (2021) 18:487–96. doi: 10.21873/cgp.20274, PMID: 33994370 PMC8240038

[24] Zhang TM Huang T Wang RF. Cross talk of chromosome instability, CpG island methylator phenotype and mismatch repair in colorectal cancer. Oncol Lett. (2018) 16:1736–46. doi: 10.3892/ol.2018.8860, PMID: 30008861 PMC6036478

[25] Liebl MC Hofmann TG. The Role of p53 Signaling in Colorectal Cancer. Cancers (Basel). (2021) 13:2125. doi: 10.3390/cancers13092125, PMID: 33924934 PMC8125348

[26] Cong B Thakur T Uribe AH Stamou E Gopinath S Maddocks O. Colon cancer cells evade drug action by enhancing drug metabolism. Oncogene. (2025). doi: 10.1038/s41388-025-03472-3, PMID: 40634495 PMC12399418

[27] Zehir A Benayed R Shah RH Syed A Middha S Kim HR. Mutational landscape of metastatic cancer revealed from prospective clinical sequencing of 10,000 patients. Nat Med. (2017) 23:703–13. doi: 10.1038/nm.4333, PMID: 28481359 PMC5461196

[28] Skoulidis F Byers LA Diao L Papadimitrakopoulou VA Tong P Izzo J. Co-occurring genomic alterations define major subsets of KRAS-mutant lung adenocarcinoma with distinct biology, immune profiles, and therapeutic vulnerabilities. Cancer Discov. (2015) 5:860–77. doi: 10.1158/2159-8290.CD-14-1236, PMID: 26069186 PMC4527963

